# Draft Genome Sequence of Janthinobacterium lividum ID1246, Isolated from a Rainbow Trout Hatchery Biofilm

**DOI:** 10.1128/MRA.00444-21

**Published:** 2021-07-15

**Authors:** Todd Testerman, Stephen R. Reichley, Stacy King, Joerg Graf

**Affiliations:** a Department of Molecular and Cell Biology, University of Connecticut, Storrs, Connecticut, USA; b Riverence, Buhl, Idaho, USA; Loyola University Chicago

## Abstract

We present a draft genome sequence of Janthinobacterium lividum strain ID1246, isolated from within a rainbow trout hatchery raceway. *Janthinobacterium* spp. are well-known producers of antimicrobial compounds. Due to the unique isolation source, this genome may yield novel biosynthetic gene clusters.

## ANNOUNCEMENT

Janthinobacterium lividum is a Gram-negative, aerobic bacterium commonly found in a broad range of soil (1), aquatic (2–4), and host-associated (5–7) biomes. Species within the genus *Janthinobacterium* have been of particular interest due to their production of bioactive secondary metabolites (6, 8). One of the best characterized of these compounds is violacein, which has antifungal (5, 6, 9), antiviral (10), antibacterial (9), and antitumoral (9, 11, 12) effects, along with imparting the quintessential violet color associated with this species (8). Interestingly, this species has been recently implicated as a potential opportunistic pathogen of rainbow trout (Oncorhynchus mykiss) (7). Here, we present a draft genome sequence of Janthinobacterium lividum ID1246, isolated from the wall of a rainbow trout hatchery raceway.

During a large-scale bacterial culturing experiment, J. lividum ID1246 was isolated from a surface swab sample of a hatchery raceway wall on R2A agar (13) incubated at 19°C. A single colony was streaked for isolation two consecutive times onto R2A agar to obtain a pure culture before being stocked at −80°C in a glycerol solution. For genome sequencing, this stock was streaked onto R2A agar, and a single colony was then inoculated into R2A broth for DNA extraction following 24 h of growth. Genomic DNA was extracted using the MasterPure DNA and RNA purification kit (Lucigen, Middleton, WI). DNA for all steps was quantified using the Qubit 1X double-stranded DNA (dsDNA) high-sensitivity (HS) assay kit (Invitrogen, Waltham, MA). A genome library was constructed using a DNA prep kit (Illumina, San Diego, CA) followed by 2 × 250-bp paired-end sequencing on a MiSeq device using a MiSeq reagent kit V2 (500 cycles) (Illumina). Following sequencing, raw reads were demultiplexed on BaseSpace and then downloaded for further processing.

CLC Genomics Workbench version 20.0.4 (Qiagen, Hilden, Germany) was used for read trimming (maximum ambiguities, 2; minimum accuracy, 95%; minimum read length, 60 bp) and *de novo* genome assembly (minimum contig length, 200; word size, 21; bubble size, 242) with default parameters. The draft genome sequence of J. lividum ID1246 was 6.35 Mb in size and was assembled into 20 contigs with an *N*_50_ value of 986 kbp. The G+C content was 62.9%, and the average genome coverage was 76× (Table 1).

**TABLE 1:** Genome summary and database accession information

Characteristic	Data for Janthinobacterium lividum ID1246
No. of raw reads	2,000,748
No. of reads in assembly	1,992,868
Genome size (Mbp)	6.35
Genome coverage (×)	76
No. of contigs	20
*N*_50_ (bp)	985,652
G+C content (%)	62.9
No. of coding sequences	5,708
No. of protein-coding sequences	5,596
No. of tRNAs	74
No. of rRNAs	6
Closest neighbor, whole genome (% dDDH)	Janthinobacterium lividum DSM 1522 (80.4)
BioProject accession no.	PRJNA720898
BioSample accession no.	SAMN18681507
SRA accession no.	SRR14245518
WGS/GenBank accession no.	JAGRZK000000000

The genome was annotated using the NCBI Prokaryotic Genome Annotation Pipeline version 5.1 (14). A total of 5,708 coding sequences were identified. Secondary metabolite gene detection was performed using antiSMASH version 5.1.2 with relaxed detection strictness (15–19). An approximately 23-kbp region was identified as containing the violacein operon. Additionally, four putative bacteriocin genes were identified along with terpene, homoserine lactone, arylpolyne, and acyl amino acid gene clusters. Species-level identification was performed using digital DNA-DNA hybridization (dDDH) using TYGS version 267 (20). The closest genome to the subject strain was Janthinobacterium lividum DSM 1522, with a dDDH of 80.4% when using the recommended d_4_ formula.

### Data availability.

The genome of J. lividum ID1246 has been deposited in DDBJ/ENA/GenBank under accession number JAGRZK000000000. The version described in this paper is the first version, JAGRZK010000000. The BioProject, BioSample, SRA, and whole-genome sequencing (WGS) accession numbers are provided in Table 1.
